# Risk factors for prolonged mechanical ventilation in patients with severe multiple injuries and blunt chest trauma: a single center retrospective case–control study

**DOI:** 10.1002/ams2.331

**Published:** 2018-01-31

**Authors:** Yasuyuki Okabe

**Affiliations:** ^1^ Division of Acute Care Surgery Chiba Emergency Medical Center Chiba Chiba Prefecture Japan

**Keywords:** Blunt chest trauma, flail chest, mechanical ventilation, rib fracture, Thoracic Trauma Severity Score

## Abstract

**Aim:**

Blunt chest trauma is common and is associated with morbidity and mortality in patients with multiple injuries, frequently requiring invasive mechanical ventilation. The aim of this study was to elucidate risk factors for prolonged mechanical ventilation (PMV).

**Methods:**

Consecutive adult patients with multiple severe injuries and blunt chest trauma who treated in Chiba Emergency Medical Center (Chiba, Japan) between January 2008 and December 2015 were enrolled in this retrospective chart‐review study. According to ventilatory time, the patients were divided into PMV (≥7 days) and shortened mechanical ventilation (SMV; <7 days) groups. Thoracic Trauma Severity Score (TTSS) was calculated. To identify risk factors for PMV, univariate and multivariate logistic analyses and receiver operating characteristic analysis were carried out.

**Results:**

Eighty‐four and 49 patients were assigned to PMV and SMV groups, respectively. Compared with the SMV group, the PMV group had a significantly larger number of fractured ribs (*P *<* *0.01), higher rate of severe Glasgow Coma Scale (GCS ≤8) (*P *<* *0.05) and flail chest (*P < *0.001), higher TTSS (*P < *0.001), or longer intensive care unit and hospital stay (both *P *<* *0.001). Logistic analysis showed that severe GCS (odds ratio [OR] = 4.6, *P *<* *0.01), flail chest (OR = 3.0, *P *<* *0.05), and TTSS (OR = 1.2; *P *<* *0.01) were independent significant risk factors. Receiver operating characteristic analyses showed that the area under the curves for TTSS, flail chest, and severe GCS were 0.74, 0.70, and 0.58, respectively. When the three factors were combined, the area under the curve increased to 0.8.

**Conclusion:**

Severe GCS (≤8), flail chest, or TTSS may be independent risk factors. Combining the three risk factors could provide high predictive performance for PMV.

## Background

Blunt chest trauma was defined as blunt chest wall injury resulting in rib fractures, lung contusion, hemothorax, pneumothorax, and others with or without life‐threatening lung injury.^1^ According to the Japan Trauma Data Bank Report 2016, blunt chest trauma is the third most common injury; injuries to the head and lower extremities are the most common.^2^

Despite progress in intensive care for severe trauma, blunt chest trauma frequently requires some ventilatory assist and is associated with significant mortality and morbidity.^3^, ^4^, ^5^, ^6^, ^7^

In multiple trauma patients, lung contusion, flail chest, and severe head injury are possible significant risk factors for mechanical ventilatory support.^8^, ^9^, ^10^, ^11^, ^12^, ^13^, ^14^ However, to our knowledge, there have been no established risk factors for prolonged mechanical ventilation (PMV) in patients with blunt chest trauma. In addition, early prediction for prolonged ventilatory support may provide useful information for planning the management of blunt chest trauma patients. The aim of the present study was to determine risk factors for PMV in patients with severe multiple injuries and blunt chest trauma.

## Methods

### Participants and study design

Chiba Emergency Medical Center (Chiba, Japan) is the sole tertiary acute critical care center in Chiba prefecture (population, 6.25 million) and provides acute care to approximately 500 severe trauma patients per year.

The institutional ethical committee provided approval for this retrospective case–control study to be undertaken at the center.

From January 2008 to December 2015, consecutive patients with blunt chest trauma treated in the Chiba Emergency Medical Center were enrolled. Exclusion criteria were: (i) died within 48 h; (ii) did not need invasive mechanical ventilation; (iii) no rib fractures; (iv) underwent surgical rib fixation; (v) Injury Severity Score (ISS) <16; (vi) less than two injury regions; (vii) age <15 years; (viii) transferred to another hospital within 5 days; (ix) mechanically ventilated for surgical procedures, not for respiratory dysfunction.

Length of mechanical ventilation was defined as the time elapsed from the initiation of ventilatory support to the accomplishment of weaning. Prolonged mechanical ventilatory support was defined as ≥7 days, based on a report that the mean length of mechanical ventilation in blunt chest trauma was 7.3 days.^15^

Thus, all patients were divided into the PMV group and the shortened mechanical ventilation (SMV; <7 days) group.

In our institution, there are always licensed trauma surgeons, orthopedic surgeons, neurologists, and intensive care unit (ICU) physicians in the hospital 24 h a day, 365 days a year, providing the same level of medical treatment. There are no residents or fellows in each shift. The initial management for all patients was carried out according to the Japan Advanced Trauma Evaluation and Care, then they received critical care in the ICU.^16^

### Data collection

Clinical data such as sex, age, systolic blood pressure, respiratory rate, Glasgow Coma Scale (GCS), and PaO_2_/FiO_2_ (P/F) ratio on admission, as well as length of hospital stay, length of ICU stay, length of mechanical ventilation, insertion of chest tube, occurrence of pneumonia (within 7 days of admission), maximum amount of fentanyl given daily, use of loxoprofen and/or acetaminophen, tracheostomy, emergency surgery, and in‐hospital death were extracted by medical chart review.

Radiographs and computed tomography scans were obtained routinely for all patients. All injuries were diagnosed by two licensed trauma surgeons on admission and checked by diagnostic radiologists within 24 h.

Severity of injury was assessed using the following scores that were coded and calculated by trained assistants and trauma surgeons. The Abbreviated Injury Scale (AIS) was coded by the AIS 90 update 98,^17^ and then the ISS and Trauma and Injury Severity Score were calculated.

The Thoracic Trauma Severity Score (TTSS) was calculated by a licensed trauma surgeon, based on five physiologic and anatomical parameters: P/F, rib fracture, pulmonary contusion, pleural involvement, and age.^9^

Flail chest was defined as three or more consecutive rib fractures, in two or more locations, consisting of a flail segment with/without paradoxical motion.

### Statistical analysis

Categorical variables are described as number (percentage), and compared by Fisher's exact test or Pearson's χ^2^‐test. Continuous variables were described as median and interquartile range, compared by the Mann–Whitney *U*‐test. Multivariate logistic analysis was carried out to determine risk factors for PMV among significant variables by univariate analysis. In addition, the number of independent variables taken into the logistic analysis was restricted to four (10 outcomes for each binary category = 49 patients in SMV group/10 = 4.9 categories). Multicollinearity was analyzed by calculating the variance inflation factor (VIF).

To evaluate the predictive performance of the risk factors determined by logistic analysis, receiver operating characteristic analysis was carried out and the area under the curve (AUC) was computed. The Youden index was calculated to determine an optimal cut‐off value.

All statistical analyses were undertaken using spss Statistics version 22 (SPSS Japan, Tokyo, Japan), and a *P*‐value <0.05 was considered significant.

## Results

**Figure 1:**
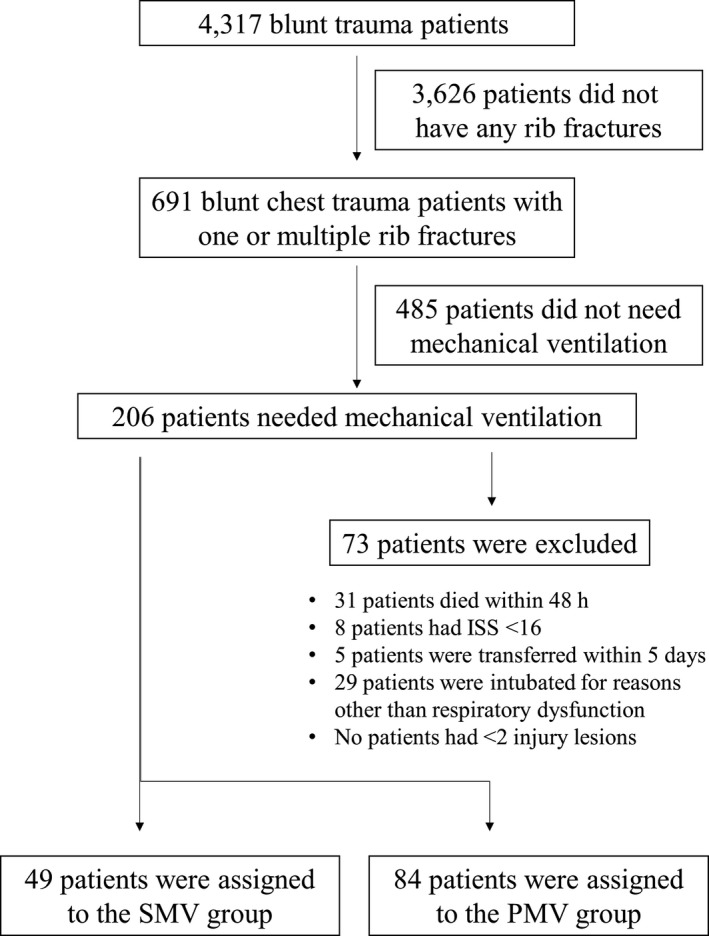
Flow diagram of patients with blunt chest trauma enrolled in this study. ISS, Injury Severity Score; PMV, prolonged mechanical ventilation; SMV, shortened mechanical ventilation.

During the study period, a total of 4,317 injured patients were admitted to our hospital. According to the exclusion criteria, 4,184 patients were excluded; 3,626 patients did not have any rib fractures, 485 patients did not need mechanical ventilation, 31 patients died within 48 hours, 8 patients had ISS <16, 5 patients were transferred within 5 days, and 29 patients were intubated for reasons other than respiratory failure. No patients had fewer than two injury lesions (Fig. 1). Of the remaining 133 patients, 84 patients were assigned to the PMV group and 49 patients were assigned to the SMV group. Medical records and radiological data were obtained from all 133 patients. Emergency surgery was carried out on 50/133 patients. All patients received invasive mechanical ventilatory support under sedation range (−2 to 1) of the Richmond Agitation Sedation Scale by a continuous infusion of dexmedetomidine or propofol with or without fentanyl. As adjunctive analgesics, loxoprofen and/or acetaminophen were given through a gastric tube at the ICU physician's discretion. Epidural analgesia was not provided for any patient.

The demographic and clinical characteristics of the patients are summarized in Table 1. The median number of mechanical ventilation days in total, and in the SMV and PMV groups were 10 days, 4 days, and 20 days, respectively. There were no significant differences between the two groups in terms of sex, age, systolic blood pressure, P/F ratio on admission, rate of pulmonary contusion, pneumothorax, hemothorax, ISS, AIS score for the head, thorax, abdomen, or extremities, maximum daily amount of fentanyl, or total number of patients receiving loxoprofen and/or acetaminophen.

**Table 1:** Demographic and clinical characteristics of patients who needed mechanical ventilation

	SMV group *n *= 49	PMV group *n *= 84	*P*‐value
Sex (male), *n* (%)	41 (84)	60 (71)	0.082
Age, years, median (IQR)	57 (42–67)	60 (45–72)	0.270
sBP ≤80 mmHg, *n* (%)	9 (5.4)	20 (28)	0.310
GCS ≤8, *n* (%)	9 (5.4)	28 (33)	0.047
P/F ratio, median (IQR)	252 (141–368)	260 (143–323)	0.550
Hemothorax, *n* (%)	33 (67)	65 (77)	0.140
Pneumothorax, *n* (%)	36 (73)	63 (75)	0.500
Lung contusion, *n* (%)	42 (86)	68 (81)	0.330
Number of fractured ribs, median (IQR)	5 (3–8)	8 (4–11)	0.001
TTSS, median (IQR)	7 (9–12)	13 (11–15)	<0.001
Flail chest, *n* (%)	11 (22)	52 (62)	<0.001
Chest tube, *n* (%)	28 (57)	58 (69)	0.120
AIS (head) ≥3, *n* (%)	21 (43)	45 (54)	0.160
AIS (abdomen) ≥3, *n* (%)	15 (21)	16 (19)	0.100
AIS (chest) ≥3, *n* (%)	43 (88)	79 (94)	0.170
AIS (extremity) ≥3, *n* (%)	21 (43)	44 (52)	0.190
ISS, median (IQR)	29 (24–41)	34 (27–38)	0.300
TRISS, median (IQR)	0.88 (0.61–0.91)	0.79 (0.56–0.89)	0.100
Maximum amount of fentanyl, mg/day, median (IQR)	0.96 (0.72–1.2)	1.2 (0.96–1.4)	0.240
Total number of patients receiving loxoprofen and/or acetaminophen, *n* (%)	29 (59)	54 (64)	0.340
Pneumonia, *n* (%)	16 (33)	38 (45)	0.110
Emergency operation, *n* (%)	15 (31)	35 (42)	0.140
Tracheostomy, *n* (%)	6 (12)	42 (50)	<0.001
Ventilation time, days, median (IQR)	4 (3–5)	20 (12–25)	<0.001
ICU stay, days, median (IQR)	6 (4–8)	16 (10–22)	<0.001
Hospital stay, days, median (IQR)	35 (16–43)	45 (32–59)	<0.001
Mortality, *n* (%)	1 (2)	13 (15)	0.011

AIS, abbreviated injury scale; GCS, Glasgow Coma Scale; ICU, intensive care; IQR, interquartile range; ISS, Injury Severity Score; PMV, prolonged mechanical ventilation; sBP, systolic blood pressure; SMV, shortened mechanical ventilation; TRISS, Trauma and Injury Severity Score; TTSS, Thoracic Trauma Severity Score.

Compared with the SMV group, the PMV group had a significantly larger number of fractured ribs (*P *= 0.001), higher rate of severe GCS (≤8) (*P *=* *0.047), higher rate of flail chest (*P *<* *0.001), and higher score of TTSS (*P *<* *0.001). The PMV group had a longer ICU stay (*P *<* *0.001) and hospital stay (*P *<* *0.001) and higher rates of tracheostomy (*P *<* *0.001) and mortality (*P *=* *0.011). All tracheostomies in both groups were carried out >7 days after ICU admission. In the SMV group, five patients could successfully wean from mechanical ventilation within 6 days and had a T‐piece installed, but they finally underwent a tracheostomy due to severe head trauma after leaving the ICU.

Multivariate logistic regression analysis showed that severe GCS (≤8) (odds ratio [OR] = 4.6; 95% confidence interval [CI], 1.2–13; *P *=* *0.003), flail chest (OR = 3.0; 95% CI, 1.1–8.2; *P *=* *0.029), or TTSS (OR = 1.2; 95% CI, 1.1–1.4; *P *=* *0.008) were independent significant risk factors for PMV. The VIF ranged from 1.0 to 1.6 for the risk factors, indicating no multicollinearities among the risk factors (Table 2).

**Table 2:** Multivariate logistic regression analysis of four variables for prolonged mechanical ventilation

	Odds ratio	95% CI	*P*‐value	VIF
GCS ≤8	4.6	1.2–13	0.003	1.0
Number of fractured ribs	1.0	0.90–1.1	0.690	1.5
Flail chest	3.0	1.1–8.2	0.029	1.6
TTSS	1.2	1.1–1.4	0.008	1.7

CI, confidence interval; GCS, Glasgow Coma Scale; TTSS, Thoracic Trauma Severity Score; VIF, variance inflation factor.

**Figure 2:**
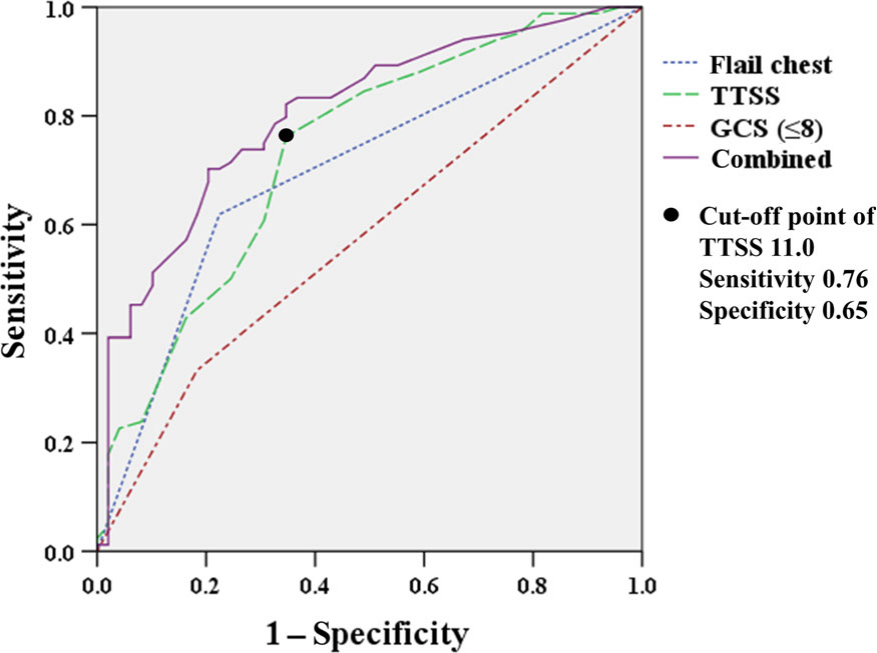
Comparison of receiver operating characteristic curves of the Thoracic Trauma Severity Score (TTSS), flail chest, and severe Glasgow Coma Scale score (GCS ≤8), alone or in combination, for prediction of prolonged mechanical ventilation.

The AUC for TTSS, severe GCS (≤8), and flail chest were 0.74 (95% CI, 0.65–0.82), 0.58 (95% CI, 0.48–0.67), and 0.70 (95% CI, 0.61–0.79), respectively. The cut‐off value for TTSS was 11 points. When the three risk factors were combined, the AUC was increased to 0.80 (95% CI, 0.73–0.88) (Fig. 2).

## Discussion

In the present study of patients with severe multiple injuries and blunt chest trauma, TTSS, severe GCS (≤8), and flail chest were determined to be independent risk factors for PMV. In addition, when the three factors were combined, the receiver operating characteristic analysis showed a high predictive performance for PMV (AUC = 0.8).

Blunt chest trauma such as rib fractures, lung contusion, pneumothorax, and hemothorax have been frequently associated with extrathoracic injuries on the head, the abdomen, and the extremities.^1^, ^8^, ^14^, ^15^, ^18^, ^19^, ^20^ Of these injuries, it is reported that the four variables, i.e. flail chest, lung contusion, the number of rib fractures, and severe head injury, may cause PMV.^8^, ^14^

Flail chest injury may be life‐threatening resulting from deformity of the chest wall and volume loss of the lung.^8^, ^21^, ^22^, ^23^ This severe injury of the chest wall may be demonstrated by inefficient breathing and respiratory suppression caused by chest pain and/or overdose of analgesics. Accordingly, the flail chest injury tends to require PMV. Dehghan *et al*. reported that 59% of flail chest patients needed ventilatory support.^8^ Huber *et al*. reported a significant correlation between flail chest injury and mechanical ventilation. The present study also indicated that flail chest is an independent risk factor for PMV.

The TTSS is a scoring system developed by Pape *et al*. combining five physiologic and anatomical parameters: PaO_2_/FiO_2_, rib fracture, pulmonary contusion, pleural involvement, and age. Each parameter is assigned a score of 0–5 according to the degree of abnormality, and the TTSS is calculated by summing the score of each parameter.^9^ To our knowledge, the present study may be the first to show that TTSS can be a risk factor with good predictive performance for PMV in patients with severe multiple injuries and blunt chest trauma. The TTSS seems to be reasonable because it includes an age parameter in addition to the physiologic and anatomical parameters. In fact, elderly patients with blunt chest trauma were reported to have higher rates of mortality and morbidity than younger patients.^24^

The four variables for the logistic regression analysis might be associated with each other. For example, considering direct external forces to the whole body in severe trauma, patients with a larger number of rib fractures tend to also have flail chest, high TTSS, or head injury. Therefore, multicollinearity analysis among the variables was carried out. The cut‐off value of VIF was determined according to the standard documented by Kutner *et al*.^25^ In the present study, a value of VIF <10 for the four variables strongly indicates no significant correlations among the factors.

Recently, an algorithm for the management of rib fractures has been published by the Western Trauma Association.^26^ In the algorithm, they focused on adequate pain control, pulmonary hygiene, and ambulation. Surgical stabilization of rib fractures (SSRF) is indicated when patients should be free of other injuries prolonging intubation or immobility. The fixation should be undertaken early (ideally within 48 h of admission), and the ribs can be fixed any time during either a video‐assisted thoracoscopy or thoracotomy. Actually, SSRF is carried out on only approximately 4% of patients with rib fractures.^27^ The results of the present study suggest that SSRF might be indicated for patients who have flail chest, are free from severe GCS (≤8), and have a higher TTSS.

The present study has several important limitations. First, the single‐center study design resulted in a small sample size. Second, our emergency medical center routinely conducts computed tomography scans on all patients before entering the ICU, which would presumably result in overdetection of clinically irrelevant lung contusion, pneumothorax, and head injury, as well as minor injuries not affecting the clinical course. Finally, epidural analgesia considered as an effective method of pain control for rib fractures was not provided due to a shortage of anesthesiologists in our medical center.

## Conclusions

Severe GCS (≤8), flail chest, or TTSS might be independent risk factors for PMV, and combining the three risk factors could provide high predictive performance.

## Disclosure

Approval of the research protocol: The present study was approved by the institutional ethical committee.

Informed consent: N/A.

Registry and registration no. of the study/trial: N/A.

Animal studies: N/A.

Conflict of interest: None declared.
